# Supplementary figures and images for: MAUI (MBI Analysis User Interface)—An image processing pipeline for Multiplexed Mass Based Imaging
Source: PLoS Comput Biol. 2021 Apr 19;17(4):e1008887. doi: 10.1371/journal.pcbi.1008887 (PMC8084329; doi:10.1371/journal.pcbi.1008887)

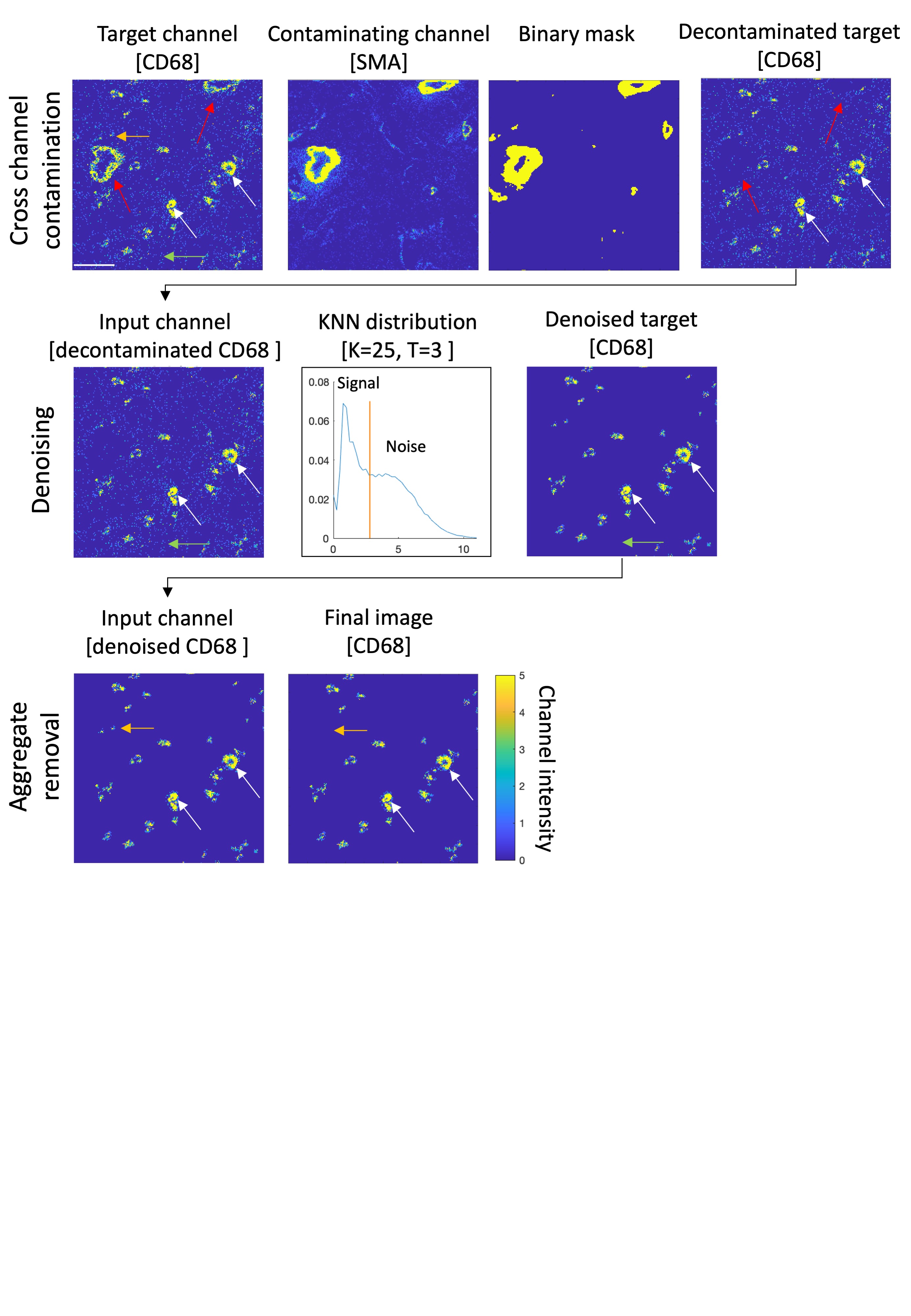

Supplement: S1 Fig — The top left panel shows an image of CD68 from an IMC dataset (Jackson et al., Nature 2020). Staining intensity is shown as a heatmap from blue (low) to high (yellow). White arrows denote real CD68 staining, as validated by coexpression of CD45. Colored arrows denote various imaging artifacts including cross-channel contamination (red), noise (green) and aggregates (orange). Each row in the image shows one stage of processing by MAUI, including removal of cross channel contamination (top), denoising (middle) and removal of aggregates (bottom) as detailed. The final image is shown in the bottom right. Scalebar equals 30μm. (JPEG) [file pcbi.1008887.s001.jpeg]

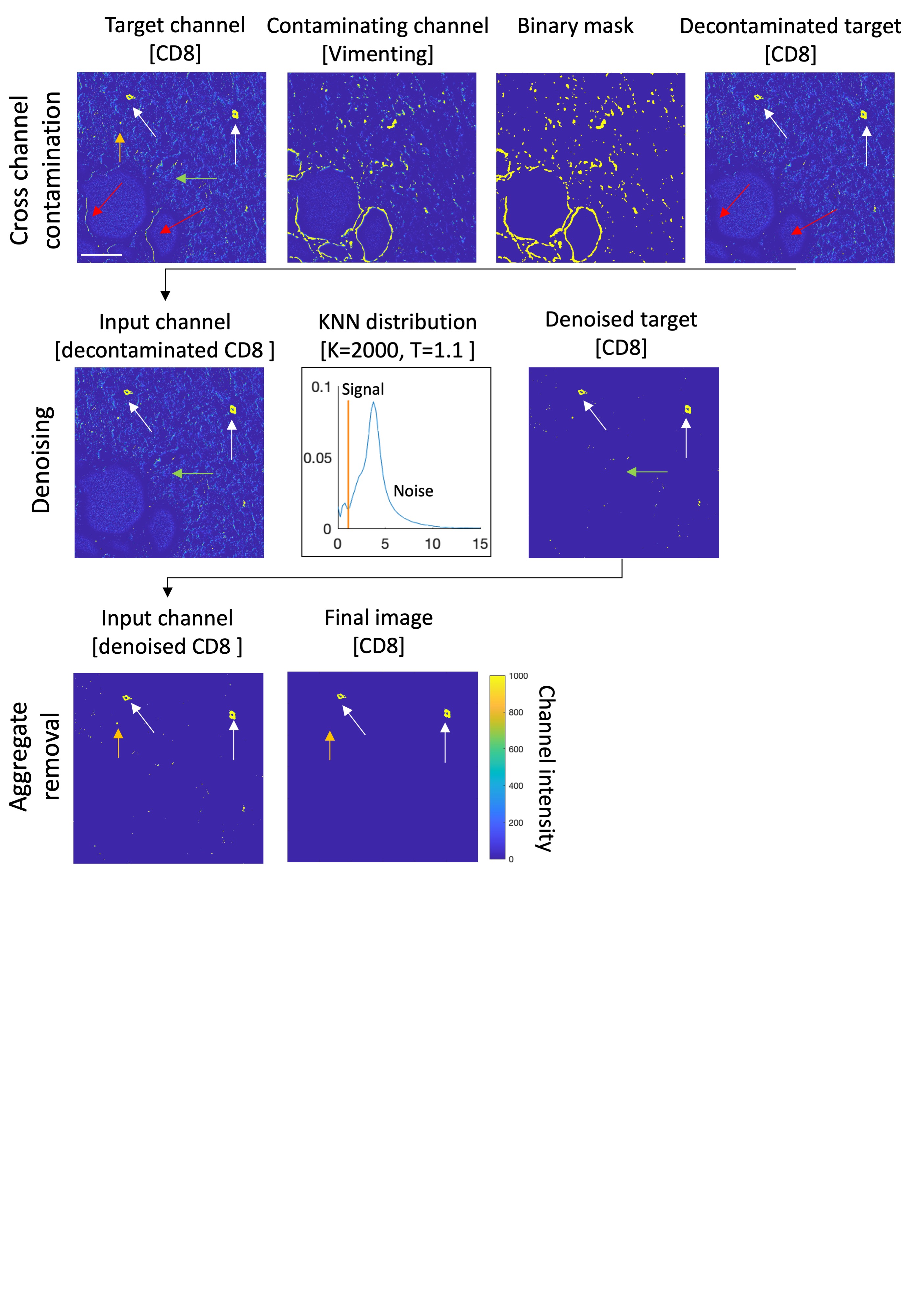

Supplement: S2 Fig — The top left panel shows an image of CD8 from a CODEX dataset (Schurch et al., Cell 2020). Staining intensity is shown as a heatmap from blue (low) to high (yellow). White arrows denote real CD8 staining, as validated by coexpression of CD3 and CD45. Colored arrows denote various imaging artifacts including cross-channel contamination (red), noise (green) and aggregates (orange). Each row in the image shows one stage of processing by MAUI, including removal of cross channel contamination (top), denoising (middle) and removal of aggregates (bottom) as detailed. The final image is shown in the bottom right. Scalebar equals 50μm. (JPEG) [file pcbi.1008887.s002.jpeg]

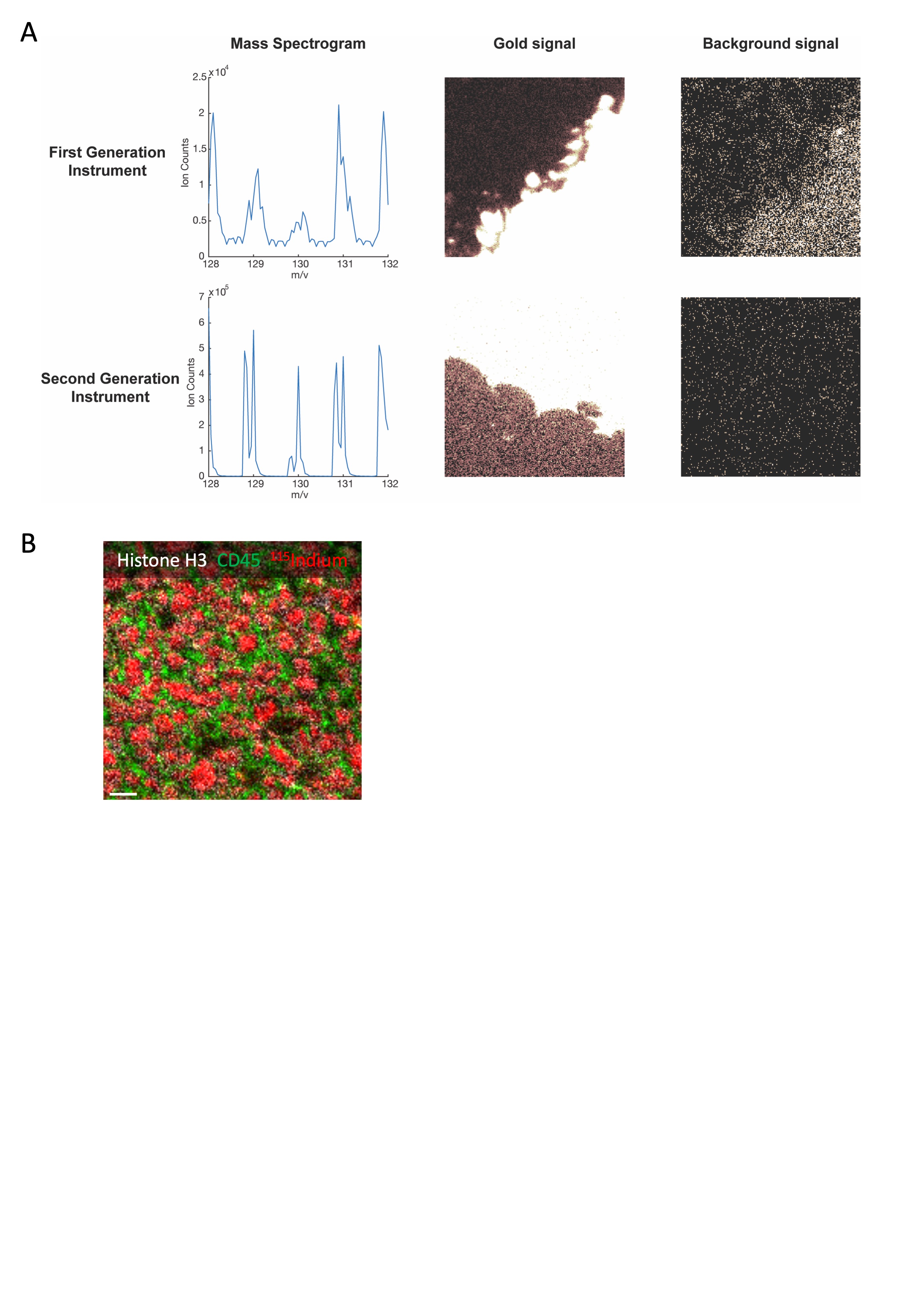

Supplement: S3 Fig — (A) Shown is an image for the mass range of 128–132, which has no labeled signal in it (left column) for the first (top) and second (bottom) generations of the instrument. In the first-generation instruments this background signal (right column) mirrors the bare slide and therefore the gold channel (middle column). In the second-generation instruments (bottom), this slide-specific background is mitigated and the background only has salt-and-pepper noise. (B) MIBI-TOF staining of human FFPE tonsil with free 115Indium and antibodies for Histone H3 and CD45 shows nuclear localization of 115Indium. Scalebar equals 10μm. (JPEG) [file pcbi.1008887.s003.jpeg]
