# Supplementary material for: MAUI (MBI Analysis User Interface)—An image processing pipeline for Multiplexed Mass Based Imaging
Source: PLoS Comput Biol. 2021 Apr 19;17(4):e1008887. doi: 10.1371/journal.pcbi.1008887 (PMC8084329; doi:10.1371/journal.pcbi.1008887)
Supplement: Attachment — Submitted filename: MAUI_pbp.pdf [file pcbi.1008887.s004.pdf]

Response to Reviewers

MAUI (MBI Analysis User Interface) - An image processing pipeline for Multiplexed Mass Based Imaging

We thank the reviewers for their in-depth consideration of our manuscript and for their insightful comments and suggestions. We were pleased to find that both reviewers were very positive about the analytical methods and potential impact of the work described in our manuscript. We thoroughly addressed the reviewers' comments below by adding additional analyses, data, supplementary figures, discussion, and references. We have revised the manuscript and believe that it is materially improved. Below we provide detailed point by point comments to the reviewers' remarks.

Reviewer #1:

Baranski and co-workers here present an interactive graphical user interface adaptation of the image processing methods originally described in Keren et al, 2018 Cell. Specifically, the software, Mass Based Imaging Analysis User Interface (MAUI), streamlines image processing from Multiplexed Ion Beam Imaging (MIBI) developed by the Angelo lab.

In addition to the MAUI software, the important work presented here also details and shows convincing examples of the results of each step performed in preliminary MIBI image processing. The authors indicate that the work can potentially extrapolate to other forms of mass spectrometry imaging, and is certainly appropriate for the scope of the journal, given the momentum in adopting various multiplexed imaging platforms in the scientific community (including the methods related to SIMS and the IMC) and the lack of alternative software that enables image cleanup without the use of scripting languages.

I have a number of minor suggestions and comments that I hope the authors can address, in addition to showcasing broad utility of MAUI.

1. In Results 2.2, the authors state tantalum as 182Ta. It would appear that 181Ta is the stable isotope of tantalum.

Thank you. Typo corrected.

2. Will the authors be able to clarify how channel 89Y would cause contaminating signals in channel 115In? It does not appear to fall into the typical +1, +16 and +17 contaminants.

We thank the reviewer for this important comment as we realize that this point was not presented clearly enough in the manuscript. We have now revised the manuscript to better emphasize that channel crosstalk can occur for various reasons, not only +1, +16 and +17 contaminants as previously described (Keren*, Bosse* et al., Science Advances 2019). For example, channel crosstalk can also occur from isotopic impurities in the metal stocks used for conjugation. Many elements naturally exist as a mixture of isotopes, and while these have been enriched to high purity for conjugation, they still have varying amounts of contaminating isotopes of the same element. For example, the element samarium has 7 naturally occurring isotopes with

an abundance ranging from 3.08% (^{144}Sm) to 26.74% (^{152}Sm). ^{152}Sm can be purified to 98.7% by electromagnetic enrichment, but will still contain small amounts of the other samarium isotopes. These residual isotopic impurities will cause channel crosstalk on MIBI.

Another reason for channel crosstalk is non-specific binding of antibodies or metal isotopes. Some metals preferentially localize to specific cellular compartments. For example, Aluminum combines with hematein to create hemalum (commonly referred to as hematoxylin), which is routinely used to stain nuclei in H&E staining (Titford et al., Biotechnic & Histochemistry 2005). Similarly, indium-hematoxylin has been used to stain nuclei in both light and electron microscopy (Gomez et al., Acta Histochemica 1991). When we stain samples with free ^{115}Indium, and visualize them using MIBI, we find that indeed ^{115}Indium localizes primarily to nuclei (Figure 1A, below). In addition, such nuclear staining does not occur when performing immunohistochemistry for HLAG (Figure 1B, below). Thus, our hypothesis is that the nuclear contamination observed for HLAG on channel 115 in figure 2 in the manuscript is a result of the nuclear localization of free indium.

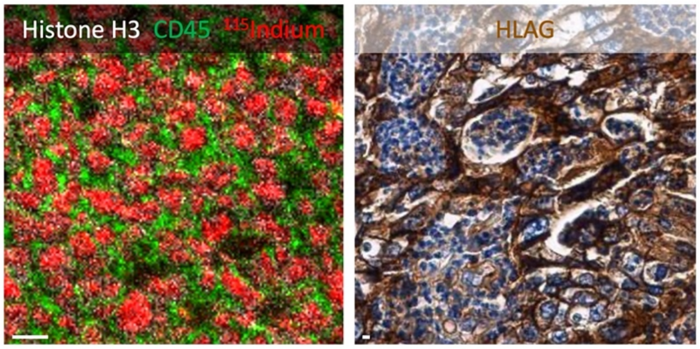

Figure 1: (A) MIBI-TOF staining of human FFPE tonsil with free ^{115}Indium and antibodies for Histone H3 and CD45 shows nuclear localization of ^{115}Indium. (B) Immunohistochemical staining of HLAG in endometrial cancer shows no nuclear staining of HLAG. Scalebars equal 10µm

Following the reviewer's comment, we have now elaborated in the text on the various reasons that may underlie cross-channel contamination (results section 2.2), and added the experiments and figure above as supplementary figure 3B.

3. It is unclear whether MAUI would allow multiple rounds of sequential contamination removal (eg removing a -1 and then a -16 on the same channel). If so, would the authors be able to elaborate further in the text?

MAUI allows multiple rounds of contamination removal. This has been added to the text.

4. In Results 2.3, the authors state that the KNN approach here "has been shown to have superior performance to other filters". It would be more convincing to have some representative images for other methods of denoising, including DBSCAN, another commonly used density-based clustering method.

DBSCAN is indeed commonly used for density-based clustering. It shares many of the underlying features as the KNN approach and has many advantages, such as not requiring pre-specification

of the number of clusters and the ability to detect outliers and noise. DBSCAN also has several disadvantages. For example, in large datasets it may not be deterministic and it encounters difficulties in data sets with large differences in densities (How et al., IEEE 2015). For tissue imaging data we find that DBscan has several disadvantages compared with the KNN approach:

1) The parameters for DBscan are less readily interpretable to non-experts. This makes it harder to tune for users.
2) DBscan is routinely used for clustering continuous data. Imaging data has integer coordinates (a pixel can be in position (1,1), but not (1.5,1.5). This imposes a non-continuous behavior of epsilon. On the other hand, the KNN effectively generates a continuous distribution, which becomes smoother as K increases (Bendall et al., Cell 2014). As such there are denoising regimes that can be reached by KNN, which cannot be reached by DBscan. In figure 2 below we present an example where an image of CD8 was denoised using the KNN approach (Fig. 2A,B) or using DBscan with scanning of the minpts parameter (Fig. 2C). We find that when minpts=2, signal is preserved, but so is noise. To eliminate noise it is necessary to increase minpts to 6, but then some deterioration in signal quality is observed.

To perform a fair comparison, the KNN also has disadvantages. For example, this algorithm scales with the absolute counts in the image and may be quite slow for images with high counts.

Importantly, the focus of this paper is to introduce a new useful tool for multiplexed image analysis, and not to claim superiority of the algorithms presented here over existing approaches. As such, following the reviewer's comment, we have removed the sentence claiming superiority from the results and simply detail the KNN algorithm and how to use it in MAUI.

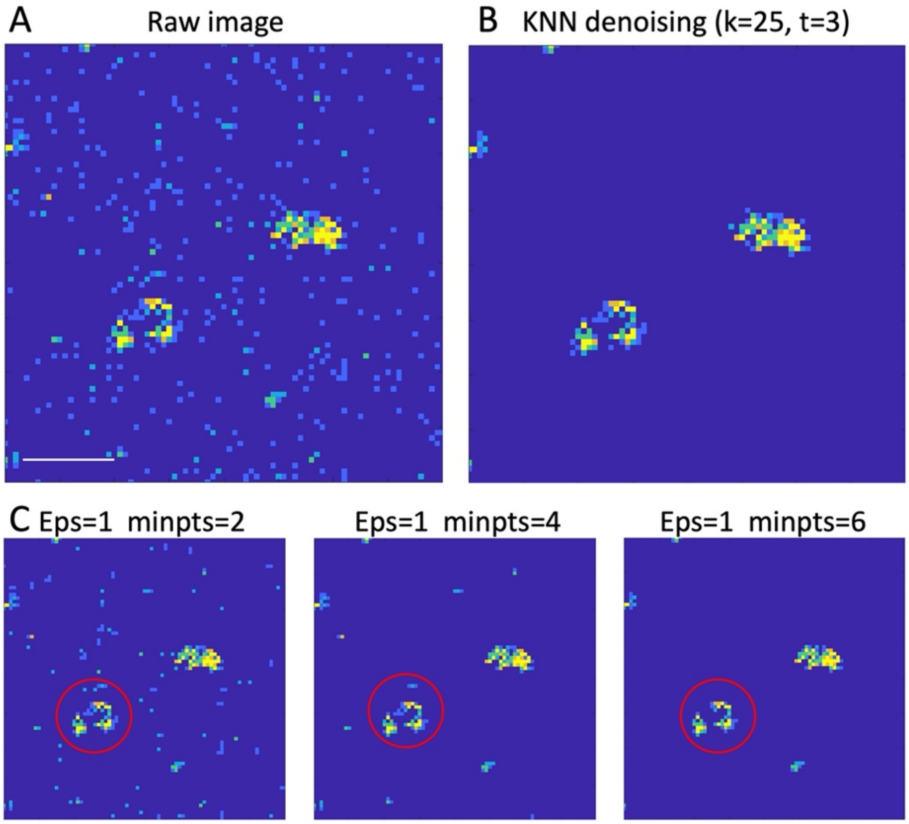

Figure 2: Comparison of KNN and DBSCAN denoising. (A) Shown is a crop from an image of CD8 staining. (B) The image in A was denoised using the KNN algorithm with k=25 and t=3. (C) The image in A was denoised using DBSCAN with epsilon=1 and increasing values of Minpts from 2 to 6. While we identify a combination of parameters for the KNN which removes noise without altering the signal, we could not find such an optimal combination for DBSCAN.

5. It is unclear how aggregation removal would be judged by a user. IE how would one decide whether large "clumps" of signal is true signal vs aggregation? It would also be useful to elaborate how the signal density of aggregates will be taken into consideration for the clean up.

Judging whether large "clumps" of signal is true signal vs aggregation can range from easy to non-trivial depending on the antibody, tissue and aggregation state. Currently, this process still depends to a large extent on experts with domain knowledge. We commonly employ the following guidelines to decide if a signal is real or an aggregate:

A. *Familiarity with the expected staining pattern of the antibody*. Whereas for some antibodies we expect small, punctate staining, which is difficult to differentiate from aggregates (e.g. Granzyme B), most of the antibodies that we use stain larger cellular structures. We use knowledge on the expected staining patterns, both intra-cellularly (e.g. nuclear vs. membrane), cellularly (e.g. expected coexpression of markers) and histologically (e.g. expected staining in some anatomical locations and not others) to decide whether a "clump of signal" is real or not. While there may be ambiguous cases, for the most part it is quite clear.
B. *Comparison between different tissues and samples*. In our experience, once an antibody vial begins to aggregate, aggregates will be observed in all samples stained with that vial. As such, if one suspects that signal is actually an aggregate, it is useful to see whether aggregates are present in this channel across additional images. MAUI allows the user to perform this evaluation quickly and conveniently.
C. *Design of specific experiments to check for aggregates*. When one is uncertain whether an antibody is aggregated, it is possible to design specific staining experiments to address this. For example, staining a negative control tissue that is not expected to express the target protein. Alternatively, one can stain serial sections and see whether the signal repeats between sections.

Following the reviewer's comment, we have now added a more detailed discussion of aggregate evaluation to the manuscript (section 4.3).

Additionally (and optionally), it would certainly strengthen the manuscript to see examples of how MAUI processing of publicly available IMC images or other mass-spec imaging data (such as ToF-SIMS, nano-SIMS or OrbiSIMS).

Following the comments by both reviewers we have now expanded the work to include an analysis of data from additional imaging modalities, including a mass-based multiplexed imaging modality (IMC) and a fluorescent-based modality (CODEX). In both modalities we identify cross-channel contamination, noise and aggregates and correct them using MAUI. We find MAUI to work well and be highly beneficial for analysis of both datasets. These analyses have now been added to the revised manuscript. Supplementary figure 1 in the manuscript (figure 3 below) demonstrates a complete analysis of an IMC image of breast cancer from Jackson et al., Nature 2020. Supplementary figure 2 in the manuscript (figure 4 below) demonstrates a complete analysis of a CODEX image of colorectal cancer from Schurch et al., Cell 2020. We thank the

reviewers for this comment, and share their belief that these analyses significantly strengthen our manuscript.

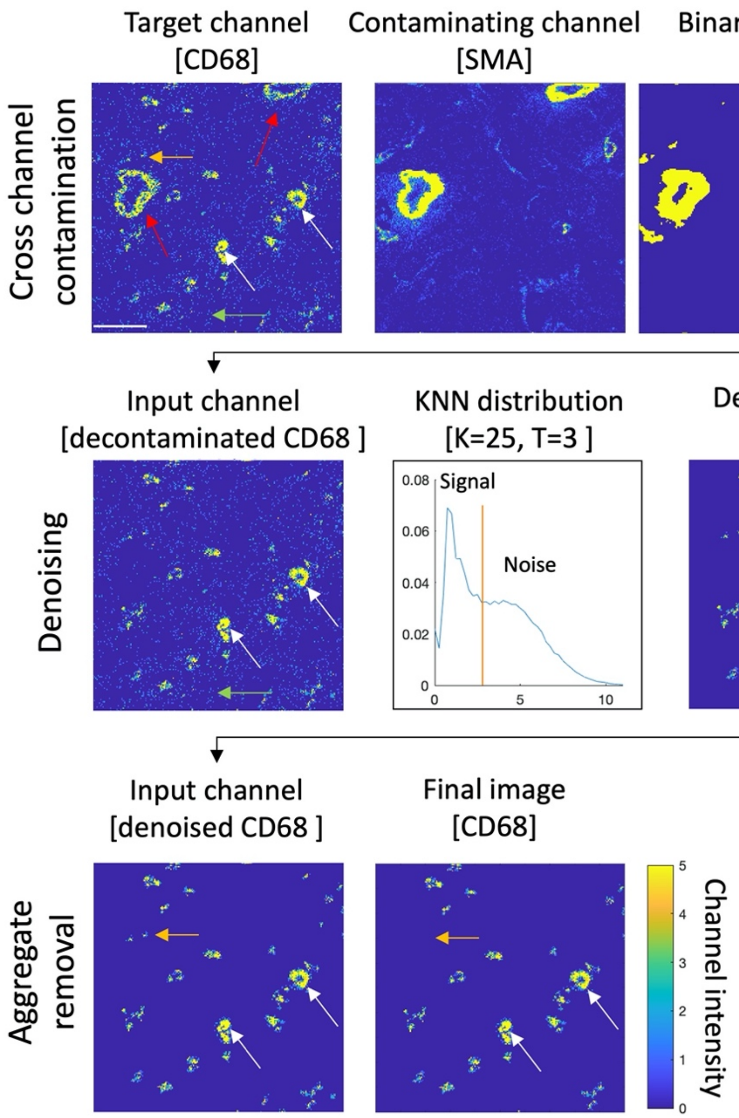

Figure 3: Analysis of IMC data using MAUI. The top left panel shows an image of CD68 from an IMC dataset (Jackson et al., Nature 2020). Staining intensity is shown as a heatmap from blue (low) to high (yellow). White arrows denote real CD68 staining, as validated by coexpression of CD45. Colored arrows denote various imaging artifacts including cross-channel contamination (red), noise (green) and aggregates (orange). Each row in the image shows one stage of processing by MAUI, including removal of cross channel contamination (top), denoising (middle) and removal of aggregates (bottom) as detailed. The final image is shown in the bottom right. Scalebar equals 30µm.

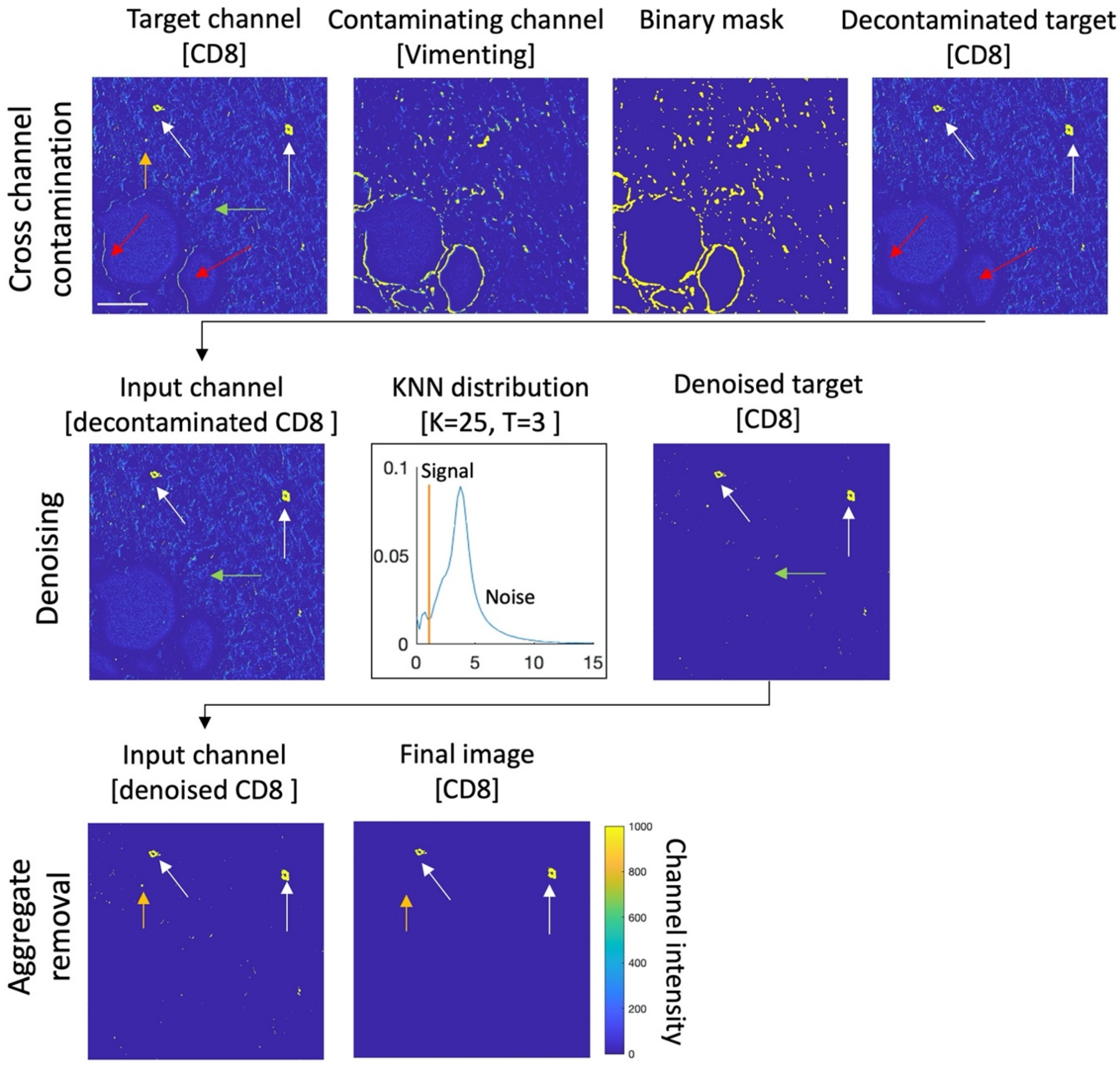

Figure 4: Analysis of CODEX data using MAUI. The top left panel shows an image of CD8 from a CODEX dataset (Schurch et al., Cell 2020). Staining intensity is shown as a heatmap from blue (low) to high (yellow). White arrows denote real CD8 staining, as validated by coexpression of CD3 and CD45. Colored arrows denote various imaging artifacts including cross-channel contamination (red), noise (green) and aggregates (orange). Each row in the image shows one stage of processing by MAUI, including removal of cross channel contamination (top), denoising (middle) and removal of aggregates (bottom) as detailed. The final image is shown in the bottom right. Scalebar equals 50µm.

Thank you for your work in putting together a very nice piece of software for the community.

Reviewer #2: The authors developed an MBI Analysis User Interface (MAUI) that facilitates the processing of image data, including the removal of channel crosstalk, noise, and antibody aggregates, and provides a software resource for efficient user data processing of data acquired by Multiplexed Ion Beam Imaging by time of flight (MIBI-TOF).

Recent developments and advancements in MIBI-TOF and Imaging Mass Cytometry (IMC) have provided innovative multiplex molecular imaging methods in the life sciences and have attracted the interest of many scientists in this field. There is no doubt that authors' contributions are important, especially for advanced lifescience researchers who are trying to adopt them.

However, after careful perusal of this manuscript, I believe that several issues need to be clarified. My main comments are as follows.

1) There is no doubt that high-dimensional imaging techniques using mass spectrometry are becoming increasingly important for histopathology, with several different instruments and methods available, including MIBI and IMC. However, since there are many non-specialist readers (especially researchers in the life sciences) who are interested in this field, I would like to see more detailed description of the instruments and methods that are currently available for users to choose from. Moreover, the instruments and methods that can be covered by MAUI should be clearly described.

Following the reviewer's comments we expanded the introduction to better introduce the field of multiplexed imaging and the various modalities used, and have added references to both original research (Moffitt et al., 2018 ; Zhu et al., 2018 ; Ke et al., 2013 ; Lee et al., 2015 ; Stahl et al., 2019 ; Wang et al., 2018 ; Chevrier et al., 2018 ; Gerdes et al., 2013 ; Gerner et al., 2012 ; Goltsev et al., 2018 ; Gut et al., 2018 ; Saga et al., 2019) and review papers dedicated to this issue (Hartmann and Bendall, 2020 ; Tan et al., 2020 ; Taube et al., 2020).
In addition, we now clearly describe the instruments and methods that can be covered by MAUI. Following the comments by both reviewers we have now expanded the work to include an analysis of data from a mass-based multiplexed imaging modality (IMC) and a fluorescent-based modality (CODEX). In both modalities we identify cross-channel contamination, noise and aggregates and correct them using MAUI. We find MAUI to work well and be highly beneficial for analysis of both datasets. These analyses have now been added to the revised manuscript. Supplementary figure 1 in the manuscript (figure 3 above) demonstrates a complete analysis of an IMC image of breast cancer from Jackson et al., Nature 2020. Supplementary figure 2 in the manuscript (figure 4 above) demonstrates a complete analysis of a CODEX image of colorectal cancer from Schurch et al., Cell 2020. We thank the reviewers for this comment, and share their belief that these analyses significantly strengthen our manuscript.

2) The reviewer is interested in whether a comparison with fluorescence microscopy images using fluorescent secondary antibodies would be useful in validating images acquired with MIBI-TOF and processed with MAUI.
In particular, it was felt that comparison with fluorescence images could clearly show whether the inherent problems in mass spectrometry caused by channel crosstalk are solved by this software. On the other hand, the problems of non-specific signals and antibody aggregation are considered to be common problems in fluorescent immunostaining methods, and it should be discussed whether it is reasonable to solve these problems with this software in MIBI-TOF images.

We thank the reviewer for this excellent suggestion. In this comment, the reviewer suggests two control experiments:

1) To show that the inherent problems in mass spectrometry caused by channel crosstalk are solved by MAUI, yielding images that are more similar to other immunostaining methods, such as immunofluorescence or immunohistochemistry.
2) To show that common problems in immunostaining methods, such as non-specific signals and antibody aggregation, can be solved by MAUI for additional imaging modalities, such as immunofluorescence.

We have now preformed both experiments (figures 4 and 5 in this document) and added them to the manuscript as supplementary figures 2 and 3, respectively.

To show that the inherent problems in mass spectrometry caused by channel crosstalk are solved by MAUI, we show a comparison between CD8 staining by immunohistochemistry (IHC, Fig. 5 top left) and by MIBI (Fig. 5, top right, green). White arrows denote true CD8 signal, staining cytotoxic T cells in the lamina propria of the gut, which is also observed by IHC. However, various imaging artifacts can be observed on the MIBI image including non-specific background, cross-channel contamination and noise (Fig. 5, bottom left, red arrows). After processing the MIBI image with MAUI, the resulting image (Fig. 5, bottom right) is strikingly similar to the image produced using IHC. Altogether processing the MIBI image with MAUI overcomes the inherent problems in mass spectrometry caused by channel crosstalk and produces staining that is similar to immunohistochemistry, as the reviewer requested. This analysis has now been added to the manuscript as Figure 2.

To show that common problems in immunostaining methods can be solved by MAUI for immunofluorescence, we analyzed a published dataset of multiplexed immunofluorescence (Schurch et al., Cell 2020). In figure 4 above we present a complete analysis of a CD8 image, including removal of cross-channel contamination, noise and aggregates. We demonstrate that MAUI is useful in processing fluorescent images and can alleviate common problems in immunostaining methods, such as non-specific signals and antibody aggregation.

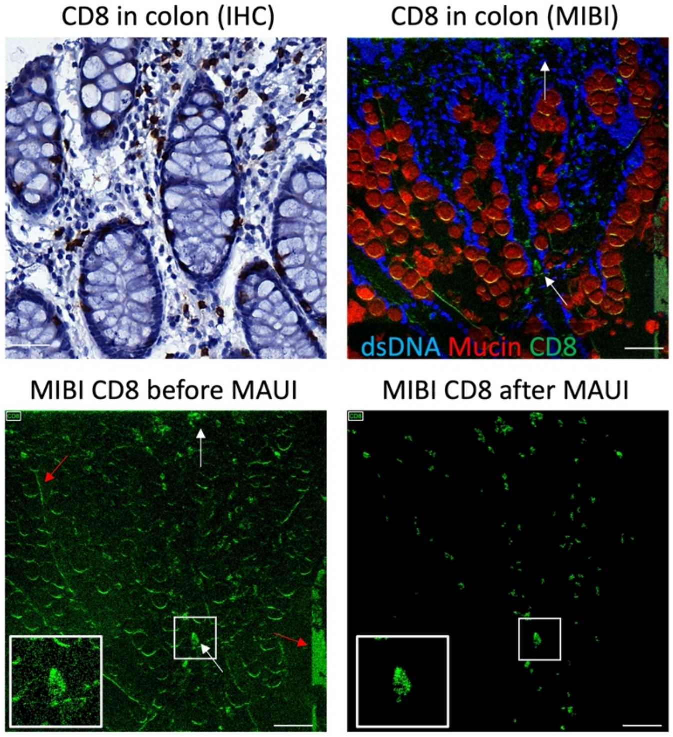

Figure 5: Validation of MAUI processing of MIBI data. Shown are serial sections from human colon stained for CD8 by IHC (top left) or MIBI (top right). White arrows indicate cytotoxic T cells in the lamina propria of the gut. Red arrows indicate imaging artifacts including background and cross-channel contamination (bottom left). After processing by MAUI (bottom right) the CD8 signal mirrors the expected histological staining pattern by IHC. Scale bar equals 40µm.
